# Estimating the Burden of Leptospirosis among Febrile Subjects Aged below 20 Years in Kampong Cham Communities, Cambodia, 2007-2009

**DOI:** 10.1371/journal.pone.0151555

**Published:** 2016-04-04

**Authors:** Sopheak Hem, Sowath Ly, Irene Votsi, Florian Vogt, Nima Asgari, Philippe Buchy, Seiha Heng, Mathieu Picardeau, Touch Sok, Sovann Ly, Rekol Huy, Bertrand Guillard, Simon Cauchemez, Arnaud Tarantola

**Affiliations:** 1 Institut Pasteur in Cambodia, Phnom Penh, Cambodia; 2 Mathematical Modelling of Infectious Diseases Unit, Institut Pasteur, Paris, France; 3 Masters of Science in Epidemiology, London School of Hygiene and Tropical Medicine, London, United Kingdom; 4 World Health Organization, Phnom Penh, Cambodia; 5 Institut Pasteur, National Reference Center and WHO Collaborating Center for Leptospirosis, Paris, France; 6 Communicable Diseases Control Department of the Ministry of Health, Phnom Penh, Cambodia; 7 National Dengue Control Program, National Center for Entomology, Parasitology, and Malaria Control, Phnom Penh, Cambodia; University of Toledo College of Medicine and Life Sciences, UNITED STATES

## Abstract

**Background:**

Leptospirosis is an emerging but neglected public health challenge in the Asia/Pacific Region with an annual incidence estimated at 10–100 per 100,000 population. No accurate data, however, are available for at-risk rural Cambodian communities.

**Method:**

We conducted anonymous, unlinked testing for IgM antibodies to *Leptospira spp*. on paired sera of Cambodian patients <20 years of age between 2007–2009 collected through active, community-based surveillance for febrile illnesses in a convenience sample of 27 rural and semi-rural villages in four districts of Kampong Cham province, Cambodia. Leptospirosis testing was done on paired serological samples negative for Dengue, Japanese encephalitis and Chikungunya viruses after random selection. Convalescent samples found positive while initial samples were negative were considered as proof of acute infection. We then applied a mathematical model to estimate the risk of fever caused by leptospirosis, dengue or other causes in rural Cambodia.

**Results:**

A total of 630 samples are coming from a randomly selected subset of 2358 samples. IgM positive were found on the convalescent serum sample, among which 100 (15.8%) samples were IgM negative on an earlier sample. Seventeen of these 100 seroconversions were confirmed using a Microagglutination Test. We estimated the probability of having a fever due to leptospirosis at 1. 03% (95% Credible Interval CI: 0. 95%–1. 22%) per semester. In comparison, this probability was 2. 61% (95% CI: 2. 55%, 2. 83%) for dengue and 17. 65% (95% CI: 17. 49%, 18. 08%) for other causes.

**Conclusion:**

Our data from febrile cases aged below 20 years suggest that the burden of leptospirosis is high in rural Cambodian communities. This is especially true during the rainy season, even in the absence of identified epidemics.

## Introduction

Leptospirosis is an endemic infectious disease found predominantly in rural regions of industrialized and developing countries worldwide [1–3]. Its prevalence is high in rural tropical areas [4–6]. Infection typically occurs during the rainy season [7–10]. Human infection is accidental, usually following exposure of non-intact skin or of mucosa to water contaminated with urine of carrier animals [11]. The natural reservoirs are mainly rodents [12–14] and other animals such as dogs, pigs and cattle [15–17]. Agricultural workers, veterinarians, butchers, fishermen, construction workers, miners, soldiers and older children / young adults are at higher risk of infection [1,18–20]. Leptospirosis infection may be asymptomatic [21,22], or may present as mild and self-limiting febrile illness resembling influenza, scrub typhus, malaria or dengue [23,24]. A small proportion of patients develop a potentially fatal illness characterized by jaundice, thrombocytopenia, haemorrhage and multiple organ dysfunctions [25–27] associated with fatality rates estimated up to 40% [28,29]. Leptospirosis is thus often underdiagnosed [13], especially in children living in areas of high Dengue endemicity [30]. Tens of millions of people are estimated to be affected by this pathogen annually [31], resulting in more than one million severe cases with approximately 60,000 deaths per year [32–34]. Early diagnosis helps guide treatment and reduce duration of hospital stay and the case fatality rate, [35]. Laboratory confirmation of leptospirosis is also challenging due to difficulties in isolating the spirochetes, in interpreting serological evidence, compounded with limitations of molecular technique, since more than 250 serovars are associated with this disease [36,37]. Antibodies are not protective and there is no cross-protective immunity to other leptospira serovars [38].

The burden of leptospirosis has been evaluated in some parts of Asia and the Pacific region [7,24,24,39–41] but detailed assessment still lacks in certain countries such as Cambodia, with a population estimated at about 13.4 million inhabitants of whom 80% are rural, working in paddy fields and small-scale cattle farming. Although leptospirosis is known to be endemic in the country [3], data on this disease remain scare. In Cambodia, leptospirosis is not a notifiable disease and laboratory testing is not available at the community level. In 2002, a study in Phnom Penh found 3% positive by MAT or PCR among children hospitalized for haemorrhagic fever [39]. Another study conducted in 2003 in Takeo provincial hospital identified 11 cases of confirmed leptospirosis in 121 inpatient and outpatient with suspected leptospirosis: 4 cases by MAT and 7 by PCR, 3 cases being positive for both MAT and PCR [42]. Another hospitalized leptospirosis study found signs of recent leptospira infection and past leptospira infection in 14.4% and 15.5% of subjects, respectively [43]. A recent study in South-Central Cambodia on undifferentiated fevers in ambulatory patients in a rural healthcare setting found 20.8% seroprevalence of IgM leptospirosis [44]. All available data in Cambodia was therefore collected in the healthcare setting.

Thanks to funding from the World Health Organization (WHO), we undertook our study to estimate the burden of Leptospirosis at community level by determining the percentage of antibody positivity and the frequency of leptospirosis seroconversion in samples taken from febrile cases in a study conducted from 2007 to 2009 in rural Cambodian communities.

## Materials and Methods

This study bore on anonymized samples already collected in past Dengue studies and stored at a biobank at the Institut Pasteur in Cambodia (IPC). Furthermore leptospirosis is a highly prevalent, endemo-epidemic disease in Cambodia. Finally, antibodies are not long—lasting and there is no cross-immunity.

For all these reasons, there was no informed subjects/guardians consent included in the protocol, which was submitted to and was approved by the National Ethic Committee for Health Research (NECHR) in Cambodia on April 8th, 2011 (#NEHCR35-2011)

### Dengue study site and population

From 2007 to 2009, inclusive, a total of 24,928 subjects (at study midpoints) aged < 20 years were recruited and prospectively followed up in the community, for varying periods of time but always including the rainy seasons which usually last from June to October. A total of 8,295 samples were collected at home during fever episodes. Serology testing was done on initial samples collected at days 0–7 and on the second sample at days 14–21 after symptom onset. Sera were aliquoted in the laboratory of the provincial hospital, transported to Institut Pasteur in Cambodia (IPC) and stored at -80°C until testing.

### Selection of the sample subset for Leptospirosis testing

**Fig 1:**
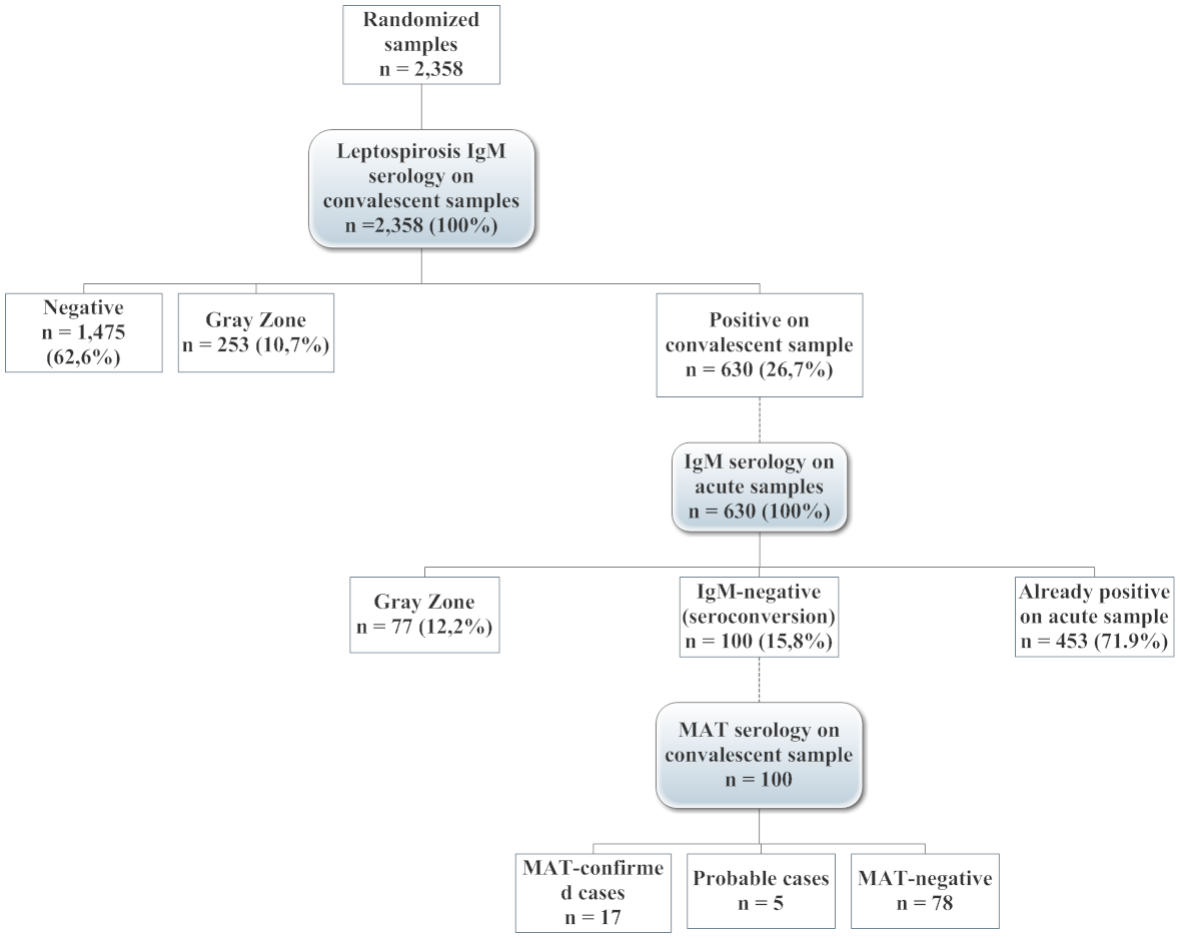
Study flowchart for febrile episodes, 2007–2009 rainy seasons, Kampong Cham province, Cambodia. Note: Dotted lines indicate when subsequent tests were performed on different samples for the same febrile episode.

Among these 8,295 samples, those already found positive by MAC-ELISA and RT-PCR for the dengue, Japanese encephalitis (JEV), Chikungunya virus, influenza, Respiratory Syncitial Virus and Human Metapneumovirus [45] were excluded, as the fevers observed were considered to be due to those virus (Fig 1). Of the remaining 7,162 samples which tested negative, 2,358 paired samples taken for fever were randomly selected and tested for leptospirosis. Furthermore, no malaria is suspected in this region and it is in a phase of elimination by 2020, as indicated in the district operational plan against malaria.

### Serology testing

#### Enzyme-Linked Immunosorbent Assay (ELISA) test

Paired acute and convalescent sera were tested for IgM anti-leptospira antibodies by using the microplate IgM-ELISA [37,46] according to the manufacturer’s instruction (Leptospira IgM ELISA, Panbio)[47] at IPC. Briefly, serum samples, triple cut-off calibrators, positive and negative control sera were diluted at 1:100 in serum diluents and 100 μL added to microwells coated by Leptospira antigens and then incubated at 37°C for 30 minutes. After washing with phosphate-buffered saline containing Tween 20 and preservative (0.1% Proclin^™^), 100 μL of HRP conjugated anti-human IgM was added and incubated for another 30 minutes at 37°C. After further washing, 100μL of tetramethylbenzidine substrate was added and incubated at room temperature for 10 minutes. We used 100μL of 1M phosphoric acid to stop the reaction. The absorbance of each well was read at wavelength of 450nm with Bio-Rad Ultramark^™^ plate reader (Bio-Rad Ultramark^™^, Microplate Imaging System). The result was expressed in units calculated by the ratio of sample absorbance to the mean cut off absorbance multiplied by 10. A positive result was defined by values >11 Panbio units and < 9 Panbio units defined negativity, as per the manufacturer’s instructions. Panbio unit values between 9 and 11 were considered in the ‘gray zone’, requiring further testing by an alternate method or renewed sampling [48]. Patients who were positive for IgM antibodies on the convalescent sample and negative on the acute sample were defined as having seroconverted. All cases of seroconversion were selected for additional testing using MAT.

#### Microagglutination test (MAT)

We first tested for IgM ELISA on the second (convalescent) sample. If positive, we then went back and tested the first [22] sample for IgM antibodies. Patients found IgM ELISA-positive on the convalescent sample and negative on the initial sample were defined as seroconverters. Convalescent samples from all seroconverters were tested by MAT to describe titers and serogroup distribution at the National Reference Center for Leptospirosis, Institut Pasteur in Paris with a panel of 24 serovars from 22 serogroups using the following antigens: serogroups Australis(serovar Australis), Autumnalis (serovar Autumnalis), Bataviae (serovar Bataviae), Ballum (serovar Castellonis), Canicola (serovar Canicola), Cynopteri (serovar Cynopteri), Celledoni (serovar Celledoni), Djasiman (serovar Djasiman), Grippotyphosa (serovar Grippotyphosa), Hebdomadis (serovar Hebdomadis), Icterohaemorrhagiae (serovar Copenhageni and icterohaemorarhagiae), Javanica (serovar Javanica), Louisiana (serovar Louisiana), Mini (serovar Mini), Panama (serovar Panama), Pomona (serovar Pomona), Pyrogenes (serovar Pyrogenes), Sejroe (serovars Hardjo and Sejroe), Sermin (serovar Sermin), Sermani (serovar Sermani), Semaranga (serovar Patoc) and Tarassovi (serovar Tarassovi). Sera were screened at a dilution of 1/50 and positive sera were titrated to endpoint using standard methods. Agglutination reactions between the serum tested and leptospira antigens were read after incubation at room temperature during 1.5 hours. High rates of agglutination of the serum with one particular antigen were used to identify the presumptive serogroup of the infecting bacterium [48]. MAT-positive leptospirosis cases were defined as having a febrile illness (consistent with leptospirosis) with evidence of seroconversion in paired sera (from a negative titre to a MAT titre ≥1/100) against any pathogenic serogroup.

### Statistical analysis

Data from the successive studies were aggregated and database field formats were standardized using Stata 11 (StataCorp, Collegestation, TX, USA). The age structure for the samples dataset was built to match the 0 < 20 age structure of the population in Kampong Cham documented for 2008 [49]. The same software package was used to randomly select the number of samples from each age group. Samples—rather than patients—were randomly selected to reduce the effect of any particular risk factor which may have artificially increased or decreased the risk of seroconversion in one patient with several febrile illnesses and therefore samples. Laboratory data were entered using Excel (Microsoft Office 2007, Microsoft, Redmond, WA., USA).

### Modeling

Date of onset and dengue testing results were available for each of the 2,358 occurrences of fever in this analysis. However, diagnosis for leptospirosis was only available for a subset of fevers. We therefore developed a statistical framework to correct for this imperfect observation and estimate individual hazards of fever caused by leptospirosis, dengue or by another cause (i.e. other than leptospirosis or dengue) from these data. The occurrence of fever during the study period was modeled with an inhomogeneous Poisson process in which each child was exposed to competitive hazards of having a fever caused by leptospirosis, dengue or another cause. Inference relied on the following assumptions: i) these hazards were constant during the three seasons under study (01/06/07-31/12/07, 01/04/08-31/12/08, 01/01/09-31/12/09) and this assumption was relaxed in a sensitivity analysis; ii) once a child was infected by dengue, the risk of further fever due to dengue was reduced by a factor 1 ‒ *ρ*_*D*_ for the rest of the season; iii) fever due to another cause did not lead to a reduction in the subsequent risk of fever; iv) fever caused by leptospirosis did not confer immunity. The probability of having a fever per semester was derived from the hazard of having a fever for presentation purposes (see S1–S3 Tables). To check the adequacy of the model, we compared predictions made by the model for simple proportions with observed values. The technical details of the model and the estimation method are presented in S1–S3 Tables.

### Ethical considerations

This study protocol was approved by the National Ethic Committee for Health Research in Cambodia NECHR on April 8^th^, 2011(# NEHCR 35–2011).

## Results

### Descriptive analysis

The febrile subjects from which samples were selected during the 3-year period were male in 1,235 (52.4%) of cases. Their median age was 9 years (range 0–19; IQR 4–13; mean 8.85 ± 5 years). Their mothers had completed primary school only in 70% of cases and had completed secondary education or higher in 8.2% of cases while 21.7% had had no schooling). In all, 2,130 (90.3%) samples originated from subjects in rural areas and 228 from semi-rural areas.

A total of 2,358 convalescent samples from 2,044 febrile subjects were initially randomly selected for leptospirosis testing (Table 1). Of these, 630 (26.7%) samples from febrile episodes in 576 subjects were found IgM positive on the convalescent serum sample (Fig 1).

**Table 1:** Year-specific and study samples enrolment and results of lpetospirosis, febrile episodes, 2007–2009 rainy seasons, Kampong Cham province, Cambodia.

Year	Number of sample selected	Study period	Months exposed	Convalescent ELISA-IgM positive (%)	ELISA- IgM seroconversion (%)	Convalescent MAT positive
2007	1,009	01/06/07–31/12/07	7	246 (24.38%)	47 (4.66%)	11 (1.09%)
2008	961	01/04/08–31/12/08	9	288 (29.96%)	36 (3.75%)	2 (0.20%)
2009	388	01/01/09–31/12/09	12	96 (24.74%)	17 (4.38%)	4 (1.03%)
Total	2,358	-	28	630 (26.71%)	100 (4.24%)	17 (0.72%)

**Fig 2:**
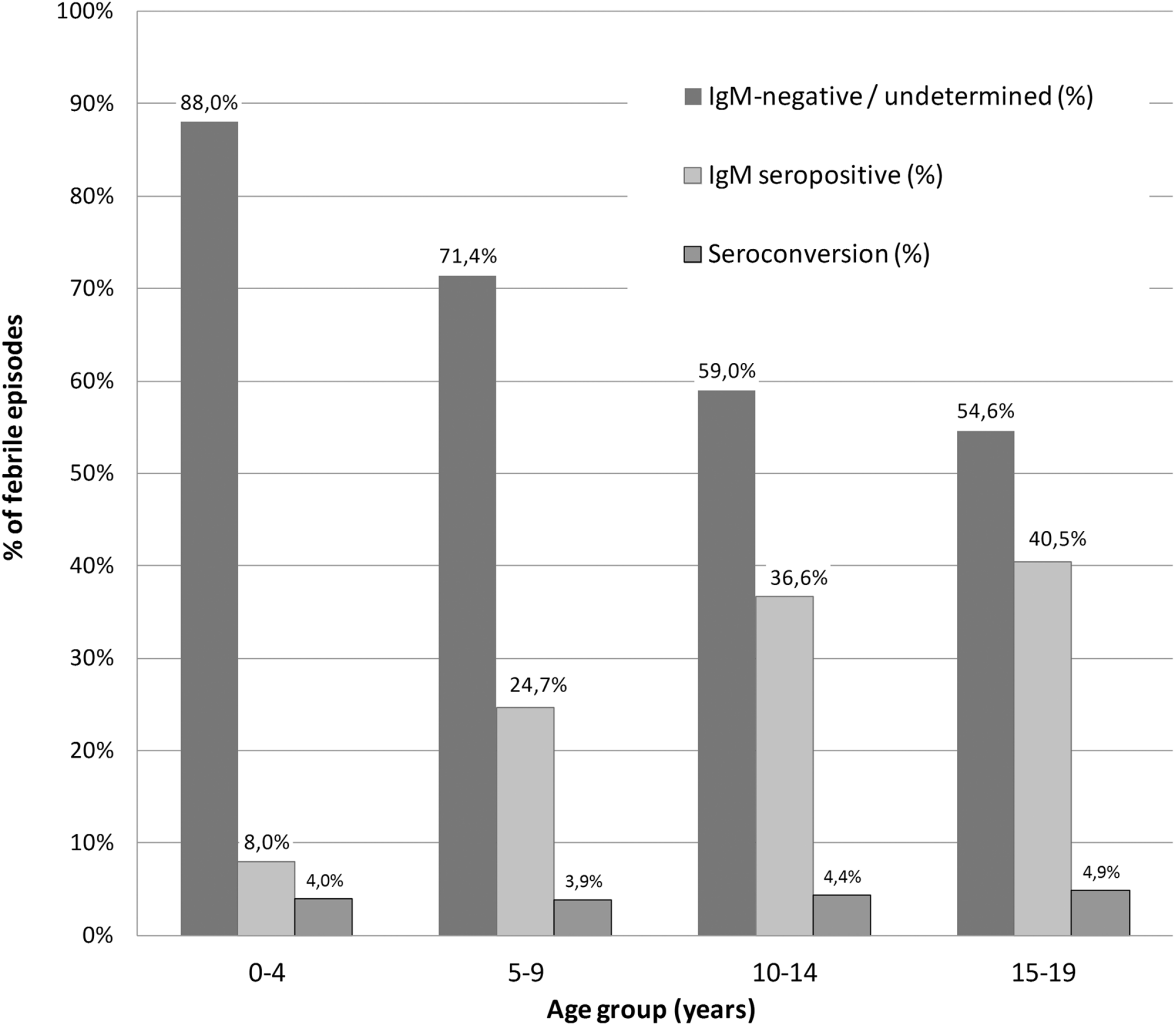
Leptospira immunological profiles by febrile patient age group, 2007–2009, Kampong Cham province, Cambodia. Percentages of samples found seronegative, seropositive (IgM positive on convalescent and initial sample) and observed seroconversion (IgM positive on convalescent sample but not initial sample).

One-hundred (15.8%) of these positive samples were negative for IgM on the acute sample and were therefore considered seroconversions in 97 patients (3 patients seroconverted twice during the study). The percentage of seroconversion in various age groups remained somewhat stable, between 4% and 4.9% (Fig 2).

Of the 100 sample pairs showing seroconversion, 17 were found MAT-positive on the convalescent sample. Ten different leptospira serogroups were identified among these 17 MAT-positive samples (Table 2).

**Table 2:** Leptospira microscopic agglutination (MAT) titres obtained in 17 positive convalescent samples, febrile episodes, 2007–2009 rainy seasons, Kampong Cham province, Cambodia.

Year	Age (y)	Gender	IgM ELISA (Panbio units)	MAT titer	Serogroup
2007	8	M	11.13	1:100	Canicola
2007	12	M	29.51	1:1600	Icterohaemorrhagiae and Sejroe
2007	9	F	32.09	1:400	Djasiman
2007	14	M	28.21	1:3200	Sejroe
2007	11	F	25.08	1:1600	Sejroe
2007	15	F	12.10	1:100	Sejroe
2007	17	M	21.32	1:800	Djasiman
2007	3	M	20.25	1:100	Tarassovi
2007	6	M	27.62	1:400	Pyrogenes
2007	16	M	24.43	1:12800	Sejroe
2007	13	M	39.47	1:1600	Djasiman
2008	15	M	32.61	1:800	Louisiana
2008	17	M	23.39	1:400	Icterohaemorrhagiae
2009	5	M	12.08	1:200	Sarmin
2009	15	M	18.92	1:200	Javanica
2009	11	M	19.63	1:200	Tarassovi
2009	9	M	17.58	1:12800	Louisiana

Patients whose successive samples showed seroconversion by ELISA IgM had a median age of 10 years (range 2 months -18 years) and were male in 57 (57%) cases. However, the 17 samples MAT-positive for leptospirosis seroconversion came from subjects with a median age of 12 years (range 3–17 years), and 14 (82.4%) were male.

### Modeling results

We estimated that the probability of having a fever caused by leptospirosis was 1.03% (95% Credible Interval CI: 0.95% -1.22%) per semester. In comparison, it was 2.61% (95% CI: 2. 55%–2.3%) for dengue and 17.65% (95% CI: 17.49% -18.08%) for other causes (Table 3). The risk of fever caused by dengue was found to be reduced by 95% (95% CI: 84%–98%) following the occurrence of a fever caused by dengue. The risk of fever caused by leptospirosis or by other causes was relatively constant across seasons, unlike the risk of fever caused by dengue which varied from 1.06% in 2008 to 4.85% in 2007.

**Table 3:** Semestrial probability of having a fever caused by leptospirosis, by dengue or by another cause. We provide the posterior mean and 95% Credible Intervals (95% CI). Technical details are presented in S1, S2 and S3 Tables.

Year	Leptospirosis	Dengue	Other Causes
2007	1.19% (1.09%, 1.54%)	4.85% (4.70%, 5.27%)	18.52% (18.27%, 19.29%)
2008	0.98% (0.88%, 1.33%)	1.06% (0.99%, 1.26%)	19.15% (18.92%, 19.84%)
2009	0.98% (0.81%, 1.48%)	2.10% (2.02%, 2.34%)	15.70% (15.47%, 16.36%)
Overall	1.03% (0.95%, 1.22%)	2.61% (2.55%, 2.83%)	17.65% (17.49%, 18.08%)

## Discussion

In our successive community studies in rural Kampong Cham, Cambodia, randomly selected samples taken in the community from febrile children and young adults in whom Dengue and other common infections were excluded were IgM positive for *Leptospira spp*. in 26.7% of cases. This is a conservative estimate as samples in the “grey zone” could not be tested further. This high proportion of positive samples can be attributed in part to the persistence of antibodies: IgM antibodies against leptospires develop approximately 4 to 7 days after the onset of symptoms [50,51]. Elevated IgM titers are most often found after 10 to 60 days [52]. IgM antibodies can be detected in 100% of patients up to the 5^th^ month following infection, in 66.7% up to the 7^th^ month and in 50% up to the 12^th^ month after the onset of symptoms [53]. In the rare event of severe leptospirosis, IgM antibodies can persist for several years [54]. The high proportion of positive samples, however, can also largely be explained by a high incidence of leptospirosis: of these positive samples from febrile children and young adults, 15.8% had initially negative samples, indirectly bearing witness to incident infection by *Leptospira spp*. and seroconversion. Our study shows that the semestrial probability of having a fever caused by leptospirosis in these Cambodian villages is at 1% of all fevers (Table 3).

Other available data from Cambodia mostly come from limited studies in hospital patients [39,42,43] and do not reflect the disease burden in the community. To our knowledge, ours is the first study to document leptospirosis in a strictly community setting in Cambodia. Our seroprevalence data are consistent with those found in ambulatory patients Cambodia [44] and other high-endemicity settings in the region. An ELISA-based community study in Southern Vietnam found an IgG prevalence of 12.8% in participants aged 7 to 12 years [41]. Studies using MAT in randomly selected healthy subjects from Southwestern province of the Mekong Delta in Vietnam found a seroprevalence ranging from 11.2% to 20.5% between the ages of 15 and 60 years, for an overall prevalence of 18.8% [55] while another study documented a seroprevalence of 23.9% in rural Lao PDR in participants aged 15 to 78 years [40]. Other MAT-based studies in a community setting conducted in Trinidad, Barbados or India found seroprevalence levels ranging between 9.5% and 23.6% among 6–8 year-olds and 20–40 year-olds, respectively [56,57].

Seroconversions were observed in 100 (15.8%) of 630 paired samples in our study. A community-based cohort study in Southern Vietnam found that 22 (10.4%) of 211 children seroconverted for IgG over a three-year period [58]. In outbreak settings, seroconversion rates may be much higher, reaching 30% of 221 children over a two-month period in India [57].

In our study, seroconversion was found mainly in samples taken from male febrile patients (57%) while MAT positive samples were found in males in 82% of cases. Other authors have shown that antibody-positive children are more likely to be male than females, perhaps due to differences in activities and lifestyles [59]. This male predominance was also found by authors in Thailand [60] or India [61].

We identified serogroups Icterohaemorrhagiae, Canicola and Sejroe which were also found during a hospital-based study in Thai children in which 19% of the non-Dengue acute febrile illnesses during the rainy season were due to Leptospirosis [30].

Our study suffers from biases and limitations which may alter our approximation of the true incidence. First, the samples were not optimally collected as the studies from which they originate were conducted over varying periods of three consecutive years, but always including the rainy season during which peak transmission is thought to occur [5,57]. Therefore the reported levels of transmission are those measured during the study months, and not the overall annual incidence to include months other than the rainy season. Water bodies, however, remain present beyond the rainy season in Cambodia, where paddy fields are remain dry for approximately 2–3 months.

Second, samples were collected only in a limited number of villages in one province, Kampong Cham. These villages, however, are located in the most populated province in the country. Given the diversity of epidemiological situations in Cambodia (i.e. forest, flooding areas, slums.), similar investigations on the causes of undifferentiated fevers are needed. This could help to better understand what is occurring in the whole country.

Third, complementary testing by MAT confirmed seroconversion in only 17% of the 100 cases found using ELISA. Low positivity by MAT might be explained by the fact that a large number of serogroups were found, and MAT may have failed to detect antibodies when specific serogroups were not used [62] and since ELISA IgM detects antibodies earlier than MAT [52]. As reported in a study in Thailand [63], some serogroups circulating in Cambodia may not have been detected in the antigen panel used in this study. The degree of correlation between MAT and ELISA also depends on the duration of disease [63]. The possibility cannot be ruled out that most of the cases detected by ELISA and MAT-negative were false positives, as ELISA kits often have low sensitivity and specificity. However, the ELISA tests we used are the ones used by other authors [52], and our percentage of positive samples were comparable. Otherwise, ELISA IgM testing detects antibodies earlier than MAT, which could also explain the observed discrepancy between the numbers of positives by using each of these two techniques.

## Conclusion

This large study identified a high burden of symptomatic leptospirosis among blood samples from febrile cases aged below 20 in rural settings in Cambodia, estimated to be about 40% of that of dengue in the region. Our results are the first truly community-based data available in this rural country, being conducted outside of the health care system. Based on these results, local physicians should be aware of leptospirosis as a frequent cause of acute febrile illness in Cambodia. Early diagnosis and appropriate antibiotic therapy should be initiated, especially in severe cases.

Further research should focus on the development of inexpensive, reliable and rapid diagnostics suitable for storage and applicable in field conditions to guide management of undifferentiated fevers. More work is also needed to document the true health and economic burden of leptospirosis in rural Cambodia, including among adults. We also hope to further our research efforts to fill the knowledge gap by focusing on asymptomatic infection and improving knowledge on the environmental, socio-economic and eco-epidemiological risk factors in different areas of the country.

## Supporting Information

S1 Table Posterior means and 95% credible intervals (95% CI) for model parameters. (TIF)

S2 Table Posterior means and 95% credible intervals (95% CI) for model parameters for each season. (TIF)

S3 Table Model validation. Observed and predicted values for simple summary statistics. Predictions are provided both for the model that assumes parameters are constant across seasons (Model 1) and the model where this assumption is relaxed (Model 2).
(TIF)
