# Supplementary figures and images for: Estimating the Burden of Leptospirosis among Febrile Subjects Aged below 20 Years in Kampong Cham Communities, Cambodia, 2007-2009
Source: PLoS One. 2016 Apr 4;11(4):e0151555. doi: 10.1371/journal.pone.0151555 (PMC4820258; doi:10.1371/journal.pone.0151555)

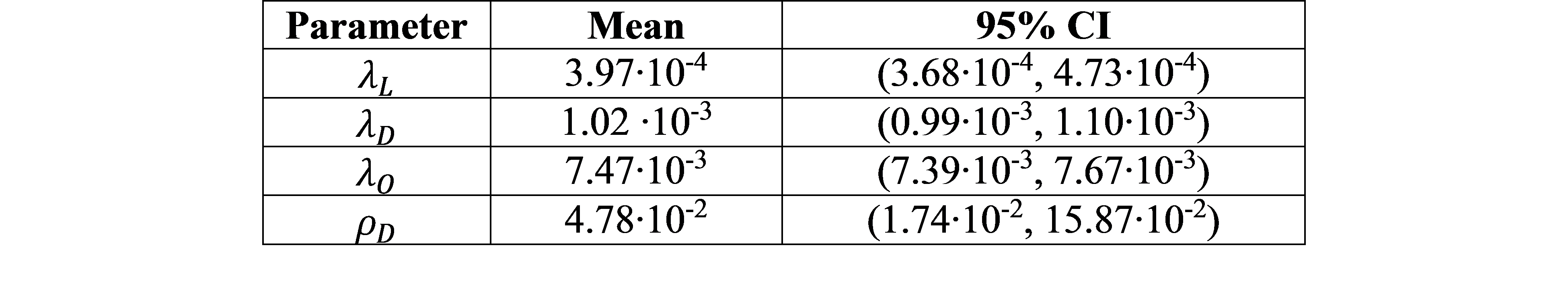

Supplement: S1 Table — (TIF) [file pone.0151555.s001.tif]

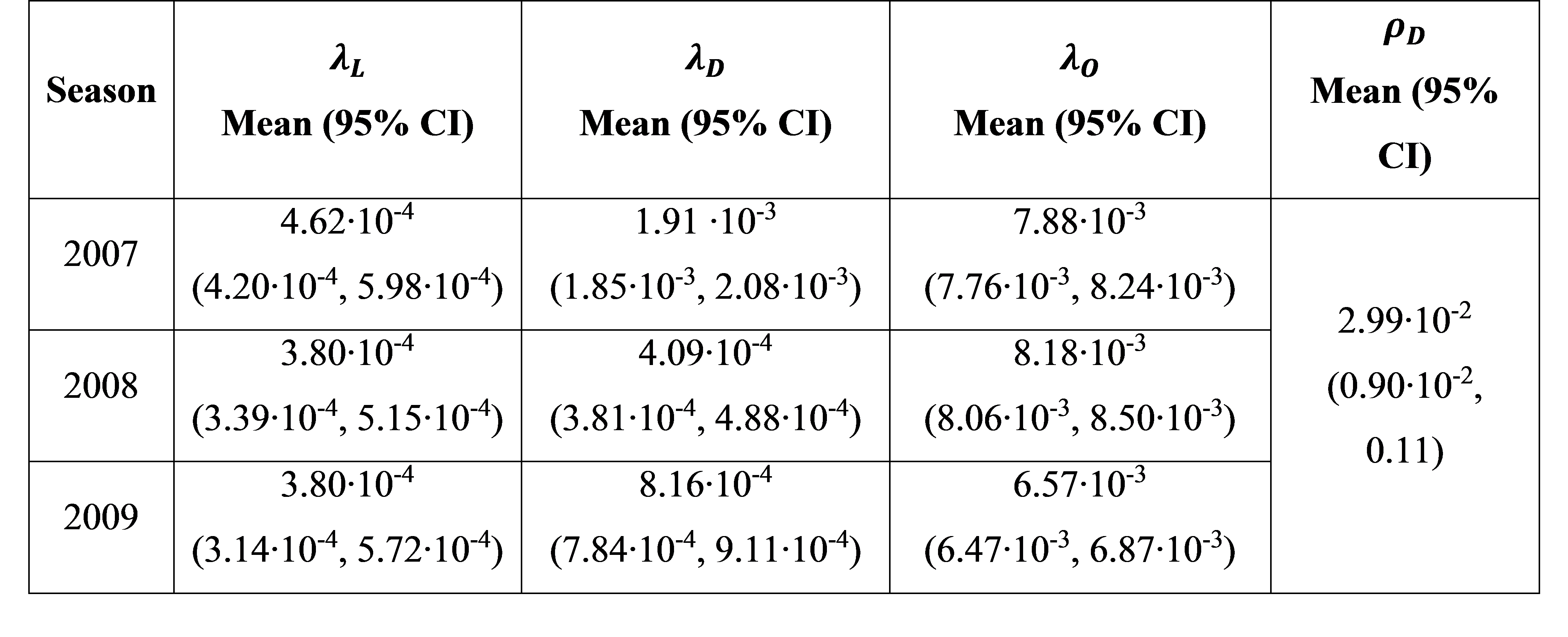

Supplement: S2 Table — (TIF) [file pone.0151555.s002.tif]

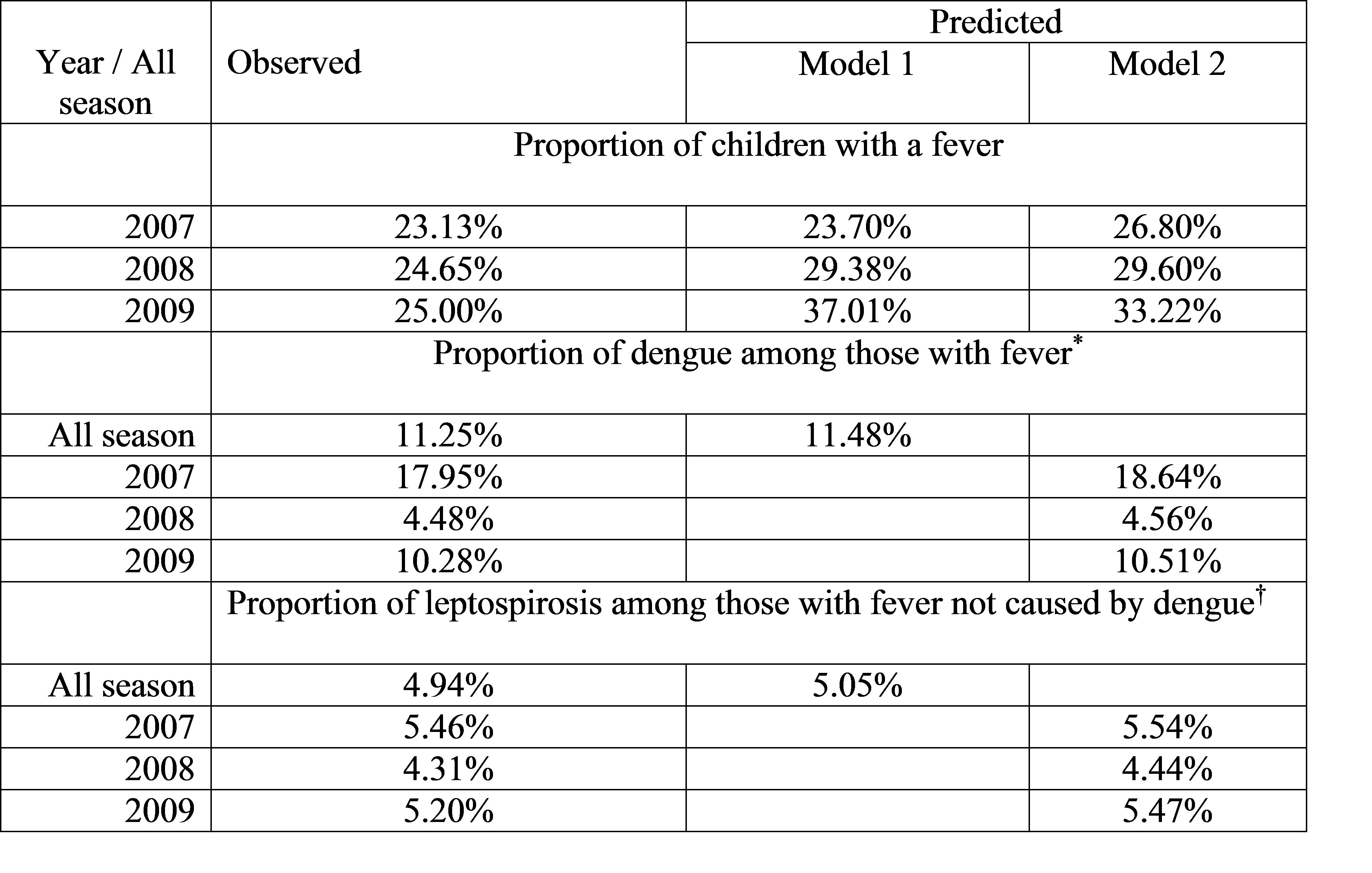

Supplement: S3 Table — Observed and predicted values for simple summary statistics. Predictions are provided both for the model that assumes parameters are constant across seasons (Model 1) and the model where this assumption is relaxed (Model 2). (TIF) [file pone.0151555.s003.tif]
